# Hepatitis Viruses Control Host Immune Responses by Modifying the Exosomal Biogenesis Pathway and Cargo

**DOI:** 10.3390/ijms231810862

**Published:** 2022-09-17

**Authors:** Eirini Karamichali, Pelagia Foka, Georgia Papadopoulou, Domniki Loukaki-Gkountara, Konstantina Andresaki, Ioannis Koskinas, Urania Georgopoulou

**Affiliations:** 1 Molecular Virology Laboratory, Hellenic Pasteur Institute, 11521 Athens, Greece; 2 2nd Department of Internal Medicine, Medical School of Athens, Hippokration General Hospital, 11521 Athens, Greece

**Keywords:** viral hepatitis, exosomes, immune response, quasi-enveloped, immunosuppression, HCC, fibrosis, cargo, HCV, HBV

## Abstract

The development of smart immune evasion mechanisms is crucial for the establishment of acute and chronic viral hepatitis. Hepatitis is a major health problem worldwide arising from different causes, such as pathogens, metabolic disorders, and xenotoxins, with the five hepatitis viruses A, B, C, D, and E (HAV, HBV, HCV, HDV, and HEV) representing the majority of the cases. Most of the hepatitis viruses are considered enveloped. Recently, it was reported that the non-enveloped HAV and HEV are, in reality, quasi-enveloped viruses exploiting exosomal-like biogenesis mechanisms for budding. Regardless, all hepatitis viruses use exosomes to egress, regulate, and eventually escape from the host immune system, revealing another key function of exosomes apart from their recognised role in intercellular communication. This review will discuss how the hepatitis viruses exploit exosome biogenesis and transport capacity to establish successful infection and spread. Then, we will outline the contribution of exosomes in viral persistence and liver disease progression.

## 1. Introduction

Liver disease arises from different causes, such as pathogens, metabolic disorders, and toxic agents [1]. The five hepatitis viruses, hepatitis A (HAV), hepatitis B (HBV), hepatitis C (HCV), hepatitis D (HDV), and hepatitis E (HEV) are collectively responsible for the occurrence of viral hepatitis, one of the most common causes of severe liver disease [2,3].

The transmission of HAV and HEV via a faecal–oral route causes self-limiting infections. Despite the low occurrence of HAV in the developed countries, where anti-HAV vaccination, proper sanitation, and clean water have made HAV outbreaks quite rare, people in low- and middle-income countries are still very much at risk, with approximately 100 million cases per year worldwide [4]. HAV does not establish persistency; however, it was shown to relapse in up to 20% of patients [5]. HEV causes waterborne outbreaks, with an estimated 20 million new cases every year in both developing and Western countries [4]. HEV was recently recognised as a zoonosis, because it is the only hepatitis virus with an intermediate animal host, usually pigs and wild boar. Although it causes acute hepatitis, there have been reports of chronicity in immunocompromised patients [6,7].

On the other hand, infection with HCV and HBV, alone or together with HDV, often leads to chronic viral hepatitis [3,8], with 59 and 296 million people suffering worldwide, respectively [9,10]. All three viruses are transmitted by bodily fluids and establish persistency accompanied by aberrant inflammation due to failure of the host to mount a successful immune response. When the liver becomes unable to heal and regenerate, hepatic fibrosis occurs, leading to the development of liver cirrhosis and, in some cases, hepatocellular carcinoma (HCC) [11]. HCC, occurring in the setting of chronic viral hepatitis, is often discovered late in its course, as the infection itself goes undiagnosed for years in most cases. Therefore, liver transplantation is the best option for the patients at this stage [12]. Interestingly, about 20–40% of HCV patients and 90% of HBV ones succeed in clearing the viruses during the acute phase of infection, depending on whether the host immune system can successfully thwart the virus-induced immune evasion [13].

The clinical manifestations of acute hepatitis include flu-like symptoms with fever and anorexia, joint pain, fatigue, and jaundice, although more than 70% of the HBV infected patients appear asymptomatic and symptoms are even more rare in acute HCV hepatitis [14,15]. The numbers of asymptomatic cases are considered to be approximately 30% and 80% for HAV and HEV, respectively; however, this could be an underestimation due to the lack of appropriate testing in the poorer countries [5,6,16].

## 2. Exosomes: The Faithful Assistants of Hepatitis Viruses

Over the last decade, extracellular vesicles (EVs), including exosomes, have gained interest in the field of cell communication as important mediators of intercellular communication in viral infections. A connection was established between viruses and EVs that contributes to viral disease progression. Several studies support that viruses may exploit the EV biogenesis route to increase their infectivity, transmissibility, and immune evasion capabilities [17,18]. For example, human immunodeficiency virus (HIV)-infected cells produce exosomes that incorporate viral proteins such as Nef [19]. In the same line, Epstein–Barr virus (EBV)-infected cells are known to release EVs containing latent membrane protein 1 (LMP-1) [20].

The focus of this review paper is to point out the importance of the interactions between exosomes and hepatitis viruses in the context of viral egress, immune evasion, immune regulation, and immunosuppression. The consequences of exosomal mediation on the development of liver disease will also be highlighted. In the next section, we provide current knowledge on the biogenesis of exosomes, their cargo, and cargo-sorting mechanisms, so that the reader comprehends the versatility of these organelles as messengers of intercellular communication and mediators of multilevel regulatory events.

### 2.1. The Making, the Taking, and the Loading

EVs originate from the endocytic pathway, and they are secreted by a variety of cells [21]. Based on their size, biogenesis mechanism, and function, they are separated in (a) apoptotic bodies (50–5000 nm in diameter), (b) microvesicles (100 nm–1 µm in diameter), and (c) exosomes (30–150 nm in diameter) [22,23,24,25]. In this review, we have decided to exclude the retrovirus-like particles (RLPs) that were previously described as indistinguishable from EVs in some cases [26,27] and are considered to be a separate EV subgroup according to the MISEV guidelines [28]. The first vesicles formed in the endocytic pathway through the invagination of the plasma membrane are the early endosomes. The biogenesis of exosomes entails vesicle budding into endosomes, followed by release from multivesicular bodies (MVBs) [24,29]. The proposed model of Fordjour and colleagues [29] suggests that both plasma and endosomal membranes participate in exosome biogenesis, indicating a new alternative concept in exosome budding and an explanation for exosomal marker heterogeneity. MVBs are late endosomes that contain multiple intraluminal vesicles (ILVs). ILVs are formed by inward invagination of the limiting endosomal membranes, a process that depends on the endosomal sorting complex required for transport (ESCRT) machinery. ILV accumulation begins when early endosomes are created and continues throughout the maturation of late endosomes. MVBs fuse either with the lysosomes or with the plasma membrane. In the first case, the contents of ILVs are degraded, whereas in the second case, the ILVs are released as exosomes in the extracellular space [30,31,32].

Exosomes are single-membrane vesicles that appear rounded and hollow under observation with transmission electron microscopy. Exosomes, isolated from body fluids or cell culture supernatants, mediate intercellular communication in normal physiology and disease carrying different cargo from recipient to target cells [33]. The exosomal cargo encompasses biomolecules such as proteins, lipids, DNAs, RNAs (mRNA, miRNAs and lncRNAs), cytokines, growth factors, etc. It is either chosen purposefully through active sorting from the cellular vicinity (endocytic vesicles, cytoplasm, and plasma membrane of the donor cell), or it is randomly engulfed during exosome formation [34]. Circulating exosomal mRNAs express new proteins in the target cells, while exosomal miRNAs and lncRNAs contribute to the post-transcriptional regulation of gene expression, thereby altering specific gene expression patterns of the target cells [35,36,37,38].

Proteins, such as tetraspanins, participate in exosome biogenesis and protein loading. Tetraspanin-enriched microdomains (TEMs) are ubiquitous specialised membrane platforms for compartmentalisation of receptors and signalling proteins in the plasma membrane [39]. It was shown that TEMs, together with tetraspanin CD81, play a key role in sorting target receptors and intracellular components toward exosomes [40]. The most commonly encountered protein content of exosomes can be divided into two groups. The first group includes proteins necessary for exosomal structural integrity, such as integral membrane and membrane-fusion-related proteins, vesicle formation proteins, and proteins of the major histocompatibility complex (MHC) classes I and II. All these molecules are derived from endosomes, plasma membrane, and cytosol of the donor cell [41,42]. The second group contains protein biomarkers commonly detected in exosomes. Exosomal biomarkers, such as ALG-2-interacting protein X (Alix) and tumour susceptibility gene 101 (TSG101), indicate the endosomal origin of exosomes. Tetraspanins CD63, CD9, and CD81 are involved in the biogenesis of exosomes [43]. Furthermore, heat shock proteins (HSPs) such as HSP70 and HSP90 are regularly found in exosomal cargo and used for the characterisation of exosomes [23]. Notably, the size of exosomes was based on a dynamic scaling model using scaling exponents, which characterise the size distributions of tumour-originating EVs [44]. Smolarz and colleagues reported that EVs isolated from the serum of healthy donors were divided in two groups of larger vesicles ranging between 50–100 nm and smaller vesicles of 20–25 nm. Western blot analysis indicated that the larger vesicles were enriched in CD63 and CD81, while CD9 and TSG101 were detected in both groups [45].

Studies have shown that the the exosomal protein cargo is not limited only to tetraspanin proteins and exosomal markers. Tetraspanines, for example, interact with proteins, such as MHC class II proteins [46], immunoglobulin superfamily member 8 (IGSF8) [47], intercellular adhesion molecule-1 (ICAM-1) [48], syndecans (SDC1–4) [49], and integrins [50]. Furthermore, CD63 interacts with different viral proteins, such as the LMP-1, leading to their secretion via exosomes [51]. Recently, it was demonstrated that exosomes derived from tumour cells secrete certain proteins known to control the immune response. Programmed death ligand 1 (PD-L1) is one of the immunosuppressor factors detected in these exosomes able to provoke T-cell anergy [52]. Signalling proteins are another group of proteins identified in exosomes, examples being the epidermal growth factor receptor (EGFR), the vascular endothelial growth factor receptor type-2 (VEGFR-2) [53], the insulin-like growth factor I receptor (IGF-1R) [54], the T-cell receptor (TCR) [55], and G protein–coupled receptors (GPCRs) [56]. Exosomes also carry a large selection of enzymes, including RNA editing enzymes, lipases, proteases, glycosyltransferases, glycosidases, and metabolic enzymes, many of which have the potential to chemically alter exosomal cargo, as reviewed in [57]. Thus, exosomes may end up carrying daughter compounds of the original intracellular molecules sorted into them upon exosomal secretion [34].

Exosomes isolated from biological fluids and various cell lines (tumoural and non-tumoural) are known to transport lipids, either as a constituent of their external lipid bilayer or as part of their internal cargo. Cholesterol, phosphatidylserine, phosphatidylcholine, esters, leukotrienes and prostaglandins, sphingomyelin, phosphatidic acid and other fatty acids of variable length and saturation, as well as various lipid species spanning up to eighteen different lipid classes have been described so far [58,59]. Exosomes were shown to contain higher quantities of certain lipid classes, such as phospholipids, cholesterol, and ceramides, in comparison with their donor cells. This is due either to the lipid sorting and loading process occurring during biogenesis of the exosomes or to de novo synthesis and modification carried out in situ by activated lipid-related enzymes that are part of the exosomal cargo [60,61]. Overall, the most recent studies emphatically propose a key role for the exosomal lipids in the regulation of the lipid metabolism of target cells, including lipid biosynthesis, transport, clearance, and degradation [62] (and references therein).

There are two different mechanisms of exosomal cargo sorting, depending on whether the ESCRT machinery is employed. This is a complex protein machinery with four distinct groups of proteins (ESCRT-0–ESCTR-III) that assists in MVB formation and vesicle budding. It is also involved in the differential cargo protein incorporation into ILVs and MVBs [63]. The function of these four protein complexes is sequential. The ESCRT-0 complex is responsible for cargo recognition, and together with the ESCRT-I, they are involved in cargo recruitment and membrane invagination. The complexes ESCRT-II and ESCRT-III are responsible for vesicle maturation and vesicle neck constriction. Finally, they carry out inward membrane scission and liberation of the fully formed ILV into the MVB lumen via the action of the vacuolar ATPase Vps4 [64,65]. Furthermore, together with the ESCRT-dependent processes, different complex lipids and proteins are involved in the biogenesis pathway of exosomes [66]. For example, ceramide participates in the initial formation of ILVs. Experiments with inactivation of ESCRT components [67] led to the identification of an alternative pathway for sorting exosomal cargo into MVBs. This mechanism depends on raft-based microdomains for the lateral segregation of cargo within the endosomal membrane. It is worth mentioning that not only do ESCRT-independent and ESCRT-dependent pathways represent alternative routes of exosomal cargo sorting, but they also act, at least in some cases, synergistically and in a cell-specific manner.

Exosomes are found in bodily fluids, including plasma [68], urine [69], and breast milk [70]. When exosomes are released from donor cells, they move freely in the extracellular space and deliver their cargo in different ways. When they reach a target cell, exosomes can fuse with their plasma membranes through a variety of mechanisms [71]. Exosomes deliver their cargo to recipient cells through attachment to their cell surface via exosomal adhesion molecules and fusion with the plasma membrane or via receptor-mediated endocytosis [33]. These interactions between target cells and exosomes lead to transfer of membrane receptors, growth factors bound on the surface of exosomes, delivery of specific proteins to target cells, and transfer of genetic material [38]. Importantly, intercellular communication is achieved through the initiation of exosome-mediated signalling cascades that can alter gene expression in target cells [72].

The origin and status of donor cells may be deduced by the presence of cell-specific exosomal markers. For example, injured liver cells secrete an increased number of exosomes enriched in carboxylesterase-1 (CES1), alcohol dehydrogenase-1 (ADH1), glutathione S-transferase, apolipoprotein A-1 (APOA-1), and albumin (ALB) [73]. Furthermore, tumour-derived exosomes carry large quantities of tumour antigens related to cellular signal transduction [74,75]. Moreover, exosomes secreted from the central nervous system (CNS) participate in physiological functions, such as maintenance of myelination, synaptic plasticity, and antigen presentation. In pathological conditions, exosomes are mainly used for the disposal of accumulating, unwanted biomolecules. For example, it was shown that in Alzheimer’s disease (AD), CNS-derived exosomes contain amyloidogenic proteins, such as hyperphosphorylated tau [76,77,78] and monomeric or oligomeric amyloid β-protein (Aβ) [79,80], while in Parkinson’s disease (PD), they can carry misfolded α-synuclein [81].

### 2.2. Hepatitis Viruses Exploit the Exosomal Biogenesis Pathway to Egress

Positive-strand RNA viruses that belong to large viral families, such as the *Flaviviridae*, *Coronaviridae*, and *Picornaviridae*, take advantage of the intracellular host cell membranes to form replication organelles, assemble viral proteins and genetic material into viral particles, and egress through cellular secretion mechanisms [82,83,84,85,86,87]. Not only does the rearrangement of membranes of the endoplasmic reticulum (ER), Golgi apparatus, and the cellular secretory pathway provide scaffolding for viral replication and transcription, but it also offers an escape vehicle for viral components from the host immune system [88,89,90], thereby facilitating immune evasion. In that context, it has lately been suggested that viruses, previously identified as non-enveloped, can be released from infected cells in a non-lytic manner enclosed in small double-layered membranous vesicles that utilise the exosomal biogenesis pathway. These viruses are now characterised as quasi-enveloped, and some hepatitis viruses seem to have adopted that route of egress [91,92,93].

Recent studies indicated that the positive-strand RNA viruses HAV and HEV, previously considered non-enveloped, have lately been found to circulate in the blood as membrane-associated, quasi-enveloped particles [93,94,95]. HAV belongs to the *Picornaviridae* family and replicates without causing cytopathic effects to host cells [96]. Interestingly, HAV may assume both forms, either as a non-enveloped virion in the bile and faeces or as a quasi-enveloped one (qeHAV) in the blood stream. Most virions present in supernatant fluids of HAV-infected human hepatoma cell cultures are also quasi-enveloped [97]. It was reported that qeHAV particles are associated with multiple components of the endolysosomal system, including exosome-associated tetraspanins, suggesting an endosomal origin for qeHAV biogenesis [94]. Feng and colleagues showed that knockdown of ESCRT-III-associated proteins inhibited the release of both enveloped and non-enveloped HAV. Conversely, the knockdown of ESCRT-0 and ESCRT-I proteins did not affect enveloped HAV release. These results suggest that enveloped HAV release is dependent on specific ESCRT-associated proteins [97]. While the exact location of HAV envelopment is not currently known, the presence of multiple endosomal proteins in extracellular qeHAV particles suggests an internal budding process, presumably involving MVBs. Consistent with this model, HAV-like particles were found in MVB-like structures in liver biopsy samples from HAV-infected owl monkeys [98].

HEV shares a similar dual lifestyle to HAV; shed in the faeces as a non-enveloped virion and circulating in the bloodstream as a quasi-enveloped (qeHEV) particle. Thus, these two phylogenetically unrelated viruses manage to accomplish this unusual envelopment in the same cellular environment. Electron microscopy revealed that HEV capsids released from infected cells via the exosomal pathway are individually wrapped in lipid membranes that resemble those of exosomes, while biogenesis of qeHEV is similar to that of exosomes [99,100]. Equally to qeHAV, the qeHEV particles contain exosomal markers, such as CD63, CD9, and CD81; epithelial cell adhesion molecule (EpCAM); and phosphatidylserine [100]. Using monoclonal antibodies generated against purified qeHEV particles produced in cell culture, a host membrane protein, trans-Golgi network protein 2 (TGOLN2), was identified on the surface of the qeHEV particle [101], providing further support for HEV budding from internal membranes. Nagashima and colleagues [102] performed siRNA experiments to show that viral particle levels released from cells depleted of exosomal secretion markers were reduced compared to controls. Finally, the HEV open reading frame-3 (ORF3) protein was essential for the interaction with the ESCRT-associated protein TSG101 and the release of the lipidated form of HEV [103,104].

**Figure 1:**
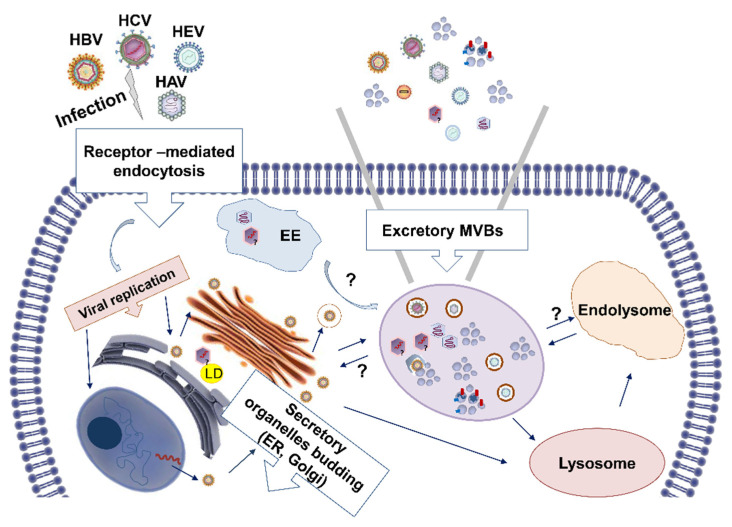
Schematic representation of the role of exosomes in the egress of hepatitis viruses. HBV, HCV, HAV, and HEV hijack the cellular endocytosis pathway to export intact virus, viral genetic material proteins packaged in exosomes. Key: EE: early endosomes; LD: lipid droplets; ?: unknown cellular events.

Apart from the hepatitis viruses that use exosomal biogenesis to egress by forming quasi-enveloped particles, HCV and HBV use exosomes to transmit whole virions or viral components. Masciopinto and colleagues identified for the first time HCV RNA in exosomes isolated from the plasma of HCV-infected patients [105]. It was later shown that HCV usurps the exosome secretory pathway to assist with viral budding [106]. Interestingly, these exosome-transmitted HCV particles were enough to establish productive infection in naïve hepatoma cells [107]. Virion formation and budding of HBV also require interaction and fusion of HBV glycoproteins with the plasma membrane and were thoroughly investigated [108,109]. Apart from this canonical route of viral transmission, HBV uses exosomes to spread. Recent studies have shown that exosomes isolated from sera of chronic HBV patients or cell cultures contain HBV DNA [110], HBV RNA [110,111], and the viral protein HBsAg [110,112]. In addition, HBV capsid and envelope proteins were found to co-localise with CD63, which was deemed necessary for HBV infectivity [113]. The following Figure 1 summarises the role of exosomes in the egress of hepatitis viruses.

Finally, several studies so far have identified a subgroup of hepatitis viruses that possess viral defective genomes (VDGs) devoid of the genes coding for the envelope proteins. The resulting defective viral particles resemble the quasi-enveloped virions. Defective viral particles were detected in the serum of infected patients. These structures enclose VDGs that can replicate but cannot be packaged into viral particles unless the infectious wild type (WT) helper virus provides the necessary missing envelope proteins, so that fully functional virions are generated. So far, VDGs have been identified in HBV [114,115], HCV [116,117], and HAV [118]. In the case of HCV, published data from our laboratory demonstrated that HCV defective genomes isolated from the sera of chronic HCV patients contributed to increased viral replication and egress. In addition to the HCV WT genome, defective HCV genomes were identified in exosomes isolated from the same sera. [117]. In chronic HBV infection, there is a correlation between the expression of defective HBV genomes (dHBV) and HBV replication. Additionally, the presence of dHBV detected in the sera of chronic HBV patients was related to liver disease severity and progression [115]. Interestingly, recent data confirmed the presence of pregenomic (pg) RNA-containing viral-like particles in the sera of chronic HBV patients and in cell cultures, which then hijacked the MVB secretory pathway for cellular egress [119]. 

## 3. Exosome-Driven Regulation of the Host Immune Responses in Viral Hepatitis

The immune responses in the liver are initiated by parenchymal cells (hepatocytes), non-parenchymal liver cells (Kupffer cells (KCs), hepatic stellate cells (HSCs)), as well as circulating immune and non-immune cells (monocytes, macrophages, dendritic cells (DCs), natural killer cells (NKCs), and platelets), which constantly infiltrate the liver. All these cells work in perfect harmony to balance immune responses against pathogen-induced immune evasion and immune suppression [120,121]. Exosomes, as intercellular mediators, participate in several physiological functions, including cell apoptosis, proliferation, differentiation, blood clotting, and tissue repair, as reviewed in [43,122,123,124,125]. Lately, it has been proposed that exosomes also play crucial roles in pathological conditions, such as tumourigenesis, tumour metastasis [126,127], and the development of neurodegenerative diseases [128]. One of the key aspects of exosome-driven disease initiation and progression is the multilevel regulation of the innate and adaptive immunity, as shown for exosomes produced by the tumour microenvironment [129,130,131,132]. This is especially true in viral infections, where the net effect of exosome-directed attenuation of the host immune response results in viral immune evasion, establishment of persistence, and eventually, chronicity.

### 3.1. Exosome-Mediated Immunomodulation of the Host Cellular Environment in Viral Hepatitis

Virus-related exosomal cargo were shown to modulate the immune response and actively promote infection establishment and chronicity, leading to liver disease progression through the transmission of exosomal immunoregulatory factors [111,133,134,135,136]. MiRNAs are among the most powerful such factors. HCV infection produces miRNA-bearing exosomes that were shown to skew innate immune responses through inhibition of NKCs by miR-122-5p, miR-146a-5p, and others [137]. Direct-acting antivirals (DAAs) are able to cure chronic HCV infection, achieving sustained virological responses (SVR) to approximately 95% of the patients [138]. These treatments have managed to reduce HCV-related exosomal miRNAs and reinstate host NKC immune responses [139]. New findings from our laboratory revealed that post-DAA treatment, chronic HCV patients at all fibrosis stages exhibited differential exosomal expression of the immunoregulatory cytokine transforming growth factor-β (TGF-β). Despite DAA-mediated HCV eradication, the presence of immunosuppressive factors in the exosomal cargo supports the notion of a remaining “viral fingerprint” that could promote liver disease in individuals suffering from advanced viral hepatitis C [134].

During HBV infection, exosomes separated from EVs by density gradients were endocytosed by DCs and macrophages following their incubation with total peripheral blood mononuclear cells (PBMCs). This led to increased PD-L1 expression on the surface of monocytes and macrophages that, in turn, weakened adaptive immunity via T-cell exhaustion. Notably, HBV reverse transcriptase inhibitory drugs were shown to alter the exosomal cargo of HBV nucleic acid species, thereby lowering its immunoregulatory potential [140].

### 3.2. Exosome-Mediated Immune Evasion of Hepatitis Viruses

Hepatitis viruses have developed mechanisms for evasion of the host immune response in order to establish successful infection [141]. Even as early as the 1980s, when the concept of exosomes was unknown, it was observed that HAV was resistant to neutralising antibodies. Experiments at the time revealed that the HAV virion was enveloped by lipids, and a vital protective relation with cell membranes was assumed [142]. Much later, exosomes released from the infected hepatic cellular milieu and characterised with the exosomal markers CD63, CD9, TSG101, and ALIX were found to transmit infective HCV components [107], masking them from the immune system. HCV-related exosomes carry a specific repertoire of miRNAs, some of which aim to make the host cell tolerate completion of the viral life cycle. The abundantly expressed liver-specific miR-122 regulates genes involved in hepatic lipid metabolism and homeostasis [143]. Importantly, a past study suggested that HCV RNA may be masked from serum neutralising antibodies when complexed with miR-122, argonaute-2 (ago2), and Hsp90 and hidden within exosomes [144]. Upon entry, miR-122 and Ago2 enhanced HCV RNA stability through base-pairing with the 5′-untranslated region (UTR) of the viral RNA, thereby leading to increased translation and HCV replication [145,146]. Interestingly, recent findings suggested that HCV-related exosomes carry large amounts of replication-competent double-stranded HCV RNAs, which can escape hepatocyte-specific toll-like receptor-3 (TLR-3)-mediated innate responses, ensuring viral propagation [147].

Next, serum exosomes from chronic HCV patients at different disease stages of HCV-associated HCC that contained CD81, one of the major HCV entry co-receptors [148], were shown to carry the virus as cargo. This either allowed the shielding of the virus from the host immune response, or held it bound to the exosomal surface through an HCV E2-exosomal tetraspanin complex. The level of enrichment of HCV in CD81+ exosomes was correlated to the HCC stage, thus, mirroring disease progression [149]. Importantly, the E2-CD81 association was demonstrated to expose E2 towards the exosomal surface [105]. It was proposed that HCV E2 protein contains a TCR inhibitory motif able to reduce T-cell activation [150]. In addition, E2-CD81 protects B-cells from activation-induced cell death, leading to weak antibody production [151], and inhibits human NKCs [152]. Neutralisation assays carried by Liu and colleagues demonstrated that a mixture of exosomal and particular HCV virus was neutralised four times less than particular HCV alone, showing that exosomes potentiate HCV immune escape against serum neutralisation [153]. Thus, the E2-CD81 association masks E2 antigenic regions against which neutralising antibodies are raised during natural infection [154]. Finally, the key sorting protein syntenin facilitates E2 exposure on the exosomal surface, thereby protecting HCV from neutralising antibodies [155].

The role of exosomes in HBV-mediated immune regulation has started to emerge from data reported by Yang and colleagues. The HBV DNA-carrying exosomes isolated from serum of chronic HBV patients and characterised by the presence of CD63 and CD81 were incubated with primary NKCs from healthy donors leading to suppressed interferon-γ (IFN-γ) production, reduced retinoic acid-inducible gene I (RIG-I) expression and inhibition of p38, and nuclear factor kappa B (NF-κB) activation. The exosome-mediated attenuation of the NKC immune response was even more pronounced upon addition of TGF-β into the NKC culture [110]. In addition, exosomal immunoregulatory miR-21 and miR-29 produced by HBV-infected hepatocytes reduced macrophagic pro-inflammatory interleukin-12 (IL-12) levels, thus, blunting macrophage activation [156]. Recently, Yang and colleagues reported the presence of a virus-encoded miRNA that proved to be an important transcriptional regulator of host and viral gene expression. HBV-miR-3, expressed in HBV-infected tissues, cells, and patient sera, was detected in HBV-containing exosomes and within HBV core particles. It plays a key role in attenuating HBV replication, translation, and virion production and keeping viral titres at very low levels so that the virus has the opportunity to bypass the host immune system and achieve persistence [157].

As far as HEV exosomes-mediated immune evasion is concerned, recent data have suggested that HEV open reading frame protein 2 (ORF2), which is the capsid protein of HEV responsible for virion assembly and encapsidation of viral RNA, was co-purified with CD63, demonstrating the dependence of HEV egress on exosomal biogenesis. Still, ORF2 remained hidden from the outer exosomal membrane, thereby protecting the viral particle from capture by ORF2 neutralising antibodies [99].

### 3.3. Exosome-Mediated Immunosuppression in Viral Hepatitis

Hepatic immunotolerant mechanisms have evolved to allow entrance of non-pathogenic antigens into the liver, protecting it from continuous injury. However, what is highly desirable under physiological conditions may lead to devastating liver disease upon viral infection. Such immunoprotective systems are exacerbated in chronic hepatitis, thereby initiating aberrant inflammation, liver fibrosis, cirrhosis, and eventually HCC development [158]. Chronic viral hepatitis sets in when the virus suppresses host immunity long enough for the infection to become persistent. Recent studies have shown that exosomal cargo is implicated in deregulated immune responses, as reviewed in neoplastic [159] and autoimmune diseases [160] characterised by aberrant inflammation. Exosomal cargo in infected hepatocytes could mirror the inflammatory state imposed by hepatitis viruses in the liver, as damaged parenchymal hepatic cells secrete exosomes containing inflammatory and immunosuppressive factors and miRNAs.

The homeobox (HOX) gene cluster expressed in the myeloid lineage, generates a class of transcripts named HOX antisense intergenic RNA (HOTAIR) lncRNAs with transcriptional regulatory functions in myelopoiesis [161]. The HOXA transcript antisense RNA myeloid-specific 1 (HOTAIRM1) is an intergenic lncRNA up-regulated during granulocyte differentiation and myeloid cell maturation [162]. Infiltrating myeloid-derived suppressor cells (MDSCs) are pathologically activated granulocytes and monocytes with potent immunosuppressive activity that inhibits T-cell functions in viral infection [163]. Although MDSCs contribute to immune homeostasis by limiting excessive inflammatory processes, their expansion may be at the expense of pathogen elimination, resulting in persistent infection [164]. During chronic HCV infection, an mRNA array analysis in myeloid cells derived from HCV patients showed concurrent up-regulation of HOTAIRM1 and its target gene HOXA1. In parallel, miR124 was inhibited and negatively correlated with HOTAIRM1 expression. HCV-containing exosomes induced the HOTAIRM1–HOXA1–miR124 axis, thereby leading to enhanced immunosuppressive MDSC functions [165]. Gene expression analysis in MDSCs isolated from HCV-infected individuals showed that there was a simultaneous up-regulation and positive correlation between expressions of the runt-related transcription factor 1 overlapping RNA (RUNXOR) and runt-related transcription factor 1 (RUNX1). Furthermore, it was demonstrated that miR-124, a downstream target of the RUNXOR–RUNX1 pathway in MDSCs, was regulated through a signal transducer and an activator of transcription 3 (STAT3) and promoted the immunosuppressive role of this pathway in MDSCs [166].

Virus-specific T-cell responses control the fate of HCV during HCV infection [167], because depletion of either CD4+ or CD8+ T-cells disturbs HCV clearance and promotes viral persistence [168,169]. Immunosuppressive mechanisms in chronic HCV infection involve high expression of several exhaustion markers, including PD-1 [170], T-cell immunoglobulin mucin-3 (Tim-3), and cytotoxic T lymphocyte-associated antigen-(CTLA-4), which restrict T-cell multifunctionality, ability to proliferate, and degranulate in response to cognate antigens [171]. Up-regulation of these negative co-stimulatory molecules is mediated by IL-2, IFN-γ, and tumour necrosis-α (TNF-α), as well as the degranulation marker CD107a, all of which prevent terminal differentiation of long-lived CD127+ memory T-cells [172,173]. Importantly, exhausted T-cells are unable to secrete antiviral cytokines and become tolerant to HCV. On top of that, T-regulatory cells (Tregs) play an important role in HCV persistent infection as they inhibit T-cell-mediated IFN-γ secretion, together with proliferation and activation of CD8+ T-cells in an antigen non-specific manner [174,175]. Recent studies demonstrate that exosomes hold a prominent role in HCV-mediated regulation of adaptive immune responses, since treatment of T-cells with CD63+ exosomes from HCV-infected hepatocytes was shown to increase a subset of CD4+-originating Tregs found in HCV-infected liver, the T-follicular regulatory cells (Tfr), in a TGF-β-specific way. In turn, Tfrs produced elevated levels of IL-10 and CTLA-4, thereby suppressing Tf helper and possibly B-cell functions [176].

HBV clearance by the host depends greatly on successful IFN-α-mediated antiviral responses. However, by using the HBV precore-p22 viral protein to block IFN-α pathway activation via inhibition of phospho-STAT1 nuclear translocation, the virus suppresses host innate immunity and achieves persistence [177]. Exosomes among other proteins carry numerous interferon-stimulated gene (ISG)-encoding proteins to recipient cells, triggering downstream signalling pertinent to the immunomodulation of innate host responses [178]. Interferon-induced transmembrane protein 2 (IFITM2) is considered to be an ISG molecule with both pro- and anti-viral activities [179]. In this case, IFITM2 was shown to act as a negative regulator of IFN-α signalling, which curbed anti-HBV responses upon external administration of IFN-α. Furthermore, IFITM2 was identified as an exosomal cargo of CD63+ exosomes derived from HBV-infected hepatocytes, which could afterwards be taken up by DCs, thereby increasing the inhibitory effect of IFITM2 on endogenous IFN-α synthesis [180]. Another study used a proteomics analysis approach to characterise the exosomal content of exosome release by an in vitro HBV replicating system. They suggested that HBV-related exosomes mediate the transmission of proteasome subunit proteins to monocytes in order to suppress pro-inflammatory IL-6 expression, thus, stressing the immunosuppressive role of exosomes in HBV infection [112].

## 4. Exosome-Mediated Disease Progression on Viral Hepatitis Background

Malignant transformation induced by oncogenic viruses, such as HCV and HBV, is a very complex procedure with multiple steps at different cellular levels. Differential protein expression, genetic instability, chronic inflammation, and immunosuppression are the major virus-orchestrated cellular events that elicit tumourigenesis in the host [181,182]. Despite the fact that hepatitis viruses are hepatotropic, extrahepatic viral replication has been suggested to exist and implies that other cell types except hepatocytes contribute to pathogenesis of viral hepatitis [183,184]. This is especially relevant to viruses capable of establishing chronicity, such as HCV, HBV, and HEV, which may replicate in PBMCs and use them as viral reservoirs [185,186,187,188,189,190,191], although this issue is still debated by some researchers. Nevertheless, extrahepatic cancers have been observed only in chronic HCV patients [192].

Exosomes are evolving into key regulators of tumourigenesis and tumour progression outcomes [193]. Exosomes isolated from cancer cell lines and exosomal fractions obtained from cancer patient plasma confirm the expression of various immunosuppressive molecules, including death receptor ligands such as FasL and TNF-related apoptosis-inducing ligand (TRAIL), checkpoint receptor ligands such as PD-L1, inhibitory cytokines, such as IL-10 and TGF-β, and non-coding RNAs [194]. These immune checkpoint molecules modulate immune responses and elicit a tolerogenic immune state able to promote carcinogenesis [195]. In turn, exosomes derived from such immunocompromised macrophages and T-cells can then infiltrate tumours and promote cancer progression, thereby closing the immunosuppressive loop [196,197,198].

Chronic HCV infection is characterised by a perpetual cycle of cell death and regeneration for the hepatocytes. Liver inflammation, oxidative stress, deregulated host homeostasis and metabolism, altered signalling, and skewed host immune responses collectively manifest as hepatic steatosis, fibrosis, cirrhosis, and hepatocarcinogenesis [199,200,201,202,203]. Exosomes foster the transition from one disease stage to the other through specific immunoregulatory exosomal cargo. For example, during an HCV-induced inflammatory state, infected hepatic cells secrete exosomal factors, such as TGF-β and the profibrotic miR-19a, resulting in HSC activation through modulation of the SOCS–STAT3 axis [133]. Similarly, miR-192, a liver injury biomarker, was identified in HCV-related exosomes and induced HSC activation into myofibroblasts through TGF-β up-regulation [204]. Interestingly, multiple miRNAs play synergistic roles in HCV-induced hepatocarcinogenesis and have been shown to be able to distinguish between liver disease progression stages in HCV infection, as reviewed in Nahand and colleagues [205].

Chronic HBV infection progresses from liver fibrosis to HCC, mostly without the cirrhotic background [206]. This is because “stealth” HBV replication possesses great oncogenic potential as it exerts constant genetic and epigenetic pressure to the host genome via insertion of the viral DNA into host chromosomes [207]. HBV viral proteins, such as HBx and HBV core, play a crucial role in the development of HBV-associated HCC through interactions with the host [208,209]. Modulation of proliferation, transformation and the hepatocytic cell cycle, induction of hepatic inflammation and oncogene expression, oxidative stress, and deregulation of the DNA damage repair mechanisms are all parts of the HBV oncogenic programme [210,211]. On its way to chronicity, HBV propagation advances by reprogramming the cell innate and adaptive immune responses towards reduced immunosurveillance, immunotolerance, unresponsiveness, and finally, exhaustion [212], eventually promoting the HCC-inducing mechanisms described above. In the light of this, late research identified an exosomal miRNA-related mechanism through which HBV-induced tumours secrete exosomal miR-142-3p that promotes M1 macrophage cell death through ferroptosis, thereby restricting tumour surveillance by infiltrating macrophages [213]. Similarly, the HBVeAg viral protein was shown to up-regulate the exosomal lncRNA MAPKAPK5-antisense RNA 1 (MAAS) in M2 macrophages, which was then implicated in increased proliferation of HBV-induced tumour cells [214].

## 5. Conclusions

**Figure 2:**
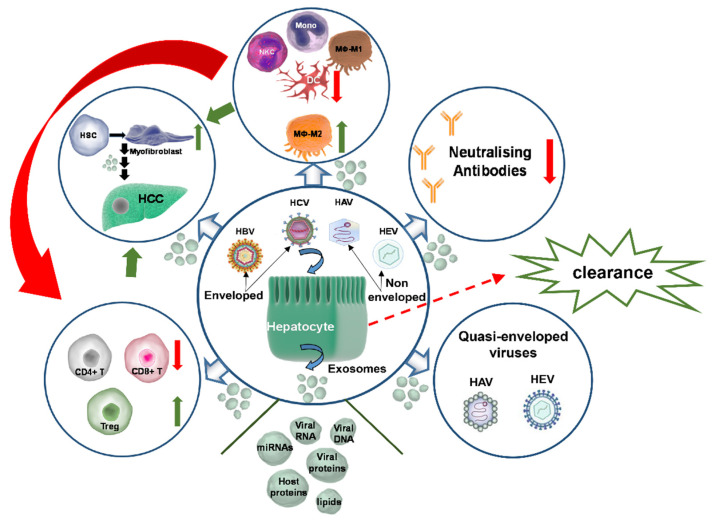
Enveloped (HCV, HBV) and non-enveloped hepatitis (HAV, HEV) viruses infect primarily hepatocytes. The non-enveloped viruses may potentially use the exosomal biogenesis pathway for egress, producing quasi-enveloped virions (bottom-right circle). All hepatitis viruses can exploit exosomes to egress and transfer their genetic material, together with viral proteins involved in replication, into permissive cells, as the exosomes grant them invisibility from host neutralising antibodies (top-right circle). However, if the host mounts efficient immune responses, as in the case of HAV and HEV, the viruses are cleared at the acute phase of infection (dashed red arrow). For viruses that achieve persistence, such as HCV and HBV, exosomes may be used for independent or consecutive (red arrow on the left) deregulation of the innate (top circle) and adaptive immune responses (bottom-left circle), leading to sustained immune evasion, immunotolerance, and immunosuppression. The exosomal cargo consists of immunoregulatory proteins, lipids, enzymes, and small RNAs (truncated cone) and may exert its immunoregulatory capacity on all immune cells, down-regulating antiviral responses whilst up-regulating tolerance. Eventually, constant immune response dysfunction, together with misdirected signalling and deregulated cellular homeostasis, lead to initiation of liver fibrosis and promote malignant transformation and HCC development (top-left circle). Key: Mono: monocytes; Mφ: macrophages; CD4+/CD8+ T: T-cells; red arrows: inhibitory action; green arrows: inducing action.

Exosomes are masters of intercellular communication and potent regulators of immune responses at all levels of immune regulation. Recently, their role in viral hepatitis has started to emerge. Hepatitis non-enveloped viruses hijack the exosomal biogenesis pathway to achieve efficient viral egress through decoration of viral capsids with exosomal membranes and components. At the same time, both enveloped and non-enveloped hepatitis viruses utilise exosomes as transporters of sensitive viral genetic material and proteins. By doing so, they effectively escape host immune responses in the form of neutralising antibodies. Exosomal cargo containing immunosuppressive viral and host proteins, as well as small RNAs, are redirected to replication permissive sites and immune sentinel cells to achieve their reprogramming, promoting persistence and chronicity. Constant immune dysfunction coupled with virus-orchestrated deregulation of cellular and molecular homeostasis ultimately lead to exacerbation of liver disease and progression to liver cancer. A summary of the key concepts discussed in this review is graphically presented in Figure 2.

## Data Availability

Not applicable.
